# Experiences of violence in daily life among adults in California: a population-representative survey

**DOI:** 10.1186/s40621-021-00367-1

**Published:** 2022-01-03

**Authors:** Garen J. Wintemute, Amanda J. Aubel, Rocco Pallin, Julia P. Schleimer, Nicole Kravitz-Wirtz

**Affiliations:** The California Firearm Violence Research Center and The Violence Prevention Research Program, University of California Davis School of Medicine, 2315 Stockton Boulevard, Sacramento, CA 95817 USA

**Keywords:** Violence, Homicide, Suicide, Firearms, Firearm ownership, Gender, Race/ethnicity, Age, Social networks, Community violence, Exposure to violence, Survey study

## Abstract

**Background:**

Research on violence exposure emphasizes discrete acute events such as direct and witnessed victimization. Little is known about the broad range of experiences of violence (EVs) in daily life. This study assesses the prevalence and patterns of distribution of 6 EVs in an adult general population.

**Methods:**

California state-representative survey administered online (English and Spanish), July 14–27, 2020. Adult (age ≥ 18 years) California resident members of the Ipsos KnowledgePanel were eligible to participate. Two EVs concerned community environments: (1) the occurrence of gunshots and shootings in the neighborhood and (2) encounters with sidewalk memorials where violent deaths occurred. Four concerned social networks: direct personal knowledge of individuals who (1) had purposefully been shot by someone else or (2) had purposefully shot themselves, and direct personal knowledge of individuals whom respondents perceived to be at risk of violence, either (3) to another person or (4) to themselves. Main outcome measures, expressed as weighted percentages with 95% confidence intervals (CIs), were the prevalence and extent (or dose) of each EV and of EVs in combination and associations between EVs and respondents’ sociodemographic characteristics and firearm ownership status.

**Results:**

Of 2870 respondents (57% completion rate), 52.3% (95% CI 49.5–55.0%) were female; mean [SD] age was 47.9 [16.9] years. Nearly two-thirds (64.6%, 95% CI 61.9–67.3%) reported at least 1 EV; 11.4% (95% CI 9.7–13.2%) reported 3 or more. Gender was not associated with the prevalence of any experience. Non-owners of firearms who lived with owners reported more extensive EVs through social networks than did firearm owners or non-owners in households without firearms. Knowledge of people who had been shot by others was most common among Black respondents, 31.0% (95% CI 20.9–43.3%) of whom knew 2 or more such persons. Knowledge of people who had shot themselves was greatest among respondents aged ≥ 60 years, but knowledge of persons perceived to be at risk of violence to themselves was greatest among respondents aged 18–29 years.

**Conclusions and relevance:**

Experiences of violence in daily life are widespread. They occur in sociodemographic patterns that differ from those for direct victimization and suggest new opportunities for research and intervention.

**Supplementary Information:**

The online version contains supplementary material available at 10.1186/s40621-021-00367-1.

## Background

Violence is epidemic in the USA. Preliminary data indicate that homicide rates rose by more than 25% nationwide in 2020 and by more than 30% in many major cities, in some cases to levels not seen in a generation (Federal Bureau of Investigation 2020; Rosenfeld et al. 2021). For cities, increases continued through the first three-quarters of 2021 (Rosenfeld et al. 2021). American civilian deaths during 2010–2019 from firearm violence (homicide and suicide) outnumber American combat fatalities from the World War II and the Vietnam Conflict combined (Centers for Disease Control and Prevention 2021; Blum and DeBruyne 2020).

Most studies of exposure to violence have focused on discrete acute events such as direct or witnessed victimization (Zimmerman and Posick 2016). There is ample evidence that these exposures can have serious, long-lasting adverse effects on the persons exposed, members of their families and social networks, and their communities, particularly when firearms are involved (Mitchell et al. 2019; Kagawa et al. 2020; Aubel et al. 2020; Leibbrand et al. 2021).

The focus of this study is on a broader range of experiences of violence (EVs) in daily life, which take many forms. Studying such experiences can shed new light on the impact of violence at the individual and community levels (Sharkey 2018). We developed measures for 6 EVs based on prior research (Mitchell et al. 2019; Kagawa et al. 2020; Aubel et al. 2020; Leibbrand et al. 2020, 2021; Aisenberg 2001; Clark et al. 2007; Zuberi 2012; Echeverria et al. 2014; Santilli et al. 2017; Smith et al. 2020) and media reports on communities highly impacted by violence, and we examined those EVs in a 2020 survey of a state-representative sample of California adults. Two EVs related to respondents’ community environments: (1) the occurrence of gunshots and shootings in the neighborhood and (2) encounters with sidewalk memorials at places where violent deaths occurred. Four EVs related to respondents’ social networks: direct personal knowledge of individuals who (1) had purposefully been shot by someone else or (2) had purposefully shot themselves, and direct personal knowledge of individuals perceived by respondents to be at risk of violence, either (3) to another person or (4) to themselves.

The peer-reviewed literature in this area is sparse. Studies of exposure to gunfire in the community have generally been on small or demographically or geographically restricted populations (Mitchell et al. 2019; Leibbrand et al. 2020, 2021; Aisenberg 2001; Clark et al. 2007; Zuberi 2012; Echeverria et al. 2014; Santilli et al. 2017). The same is true of studies of witnessing a shooting or knowing someone who had been a victim of firearm violence (Leibbrand et al. 2021; Clark et al. 2007; Santilli et al. 2017; Smith et al. 2020). To our knowledge, encounters with sidewalk memorials and knowledge of persons perceived to be at risk of violence have not been studied.

The aims of this study are to document the prevalence and extent (or dose: number of memorials encountered, number of persons known) of experiences of violence in a general-population sample and to identify associations between those experiences and respondents’ sociodemographic characteristics and firearm ownership status. We also estimate, for California, the population-level burden of these experiences.

## Methods

This study reports findings from the 2020 iteration of the California Safety and Well-being Survey (CSaWS 2020). The survey methods and analytic approach for CSaWS 2020 were described in the project’s initial report (Kravitz-Wirtz et al. 2021b) and are summarized here. CSaWS 2020 was designed to be conducted during the COVID-19 pandemic and was administered online from July 14 to July 27, 2020—during the first summer surge of COVID-19 cases in California and the USA. The survey was approved by the University of California Davis Institutional Review Board and is reported following American Association for Public Opinion Research guidelines (American Association for Public Opinion Research 2020).

The survey was administered by the research firm Ipsos (2020), and the study population was drawn from the Ipsos KnowledgePanel, an online research panel also used in the 2018 iteration of CSaWS (Kravitz-Wirtz et al. 2020; Schleimer et al. 2020a, b; Tomsich et al. 2020) and other studies related to firearm ownership (Azrael et al. 2015; Salhi et al. 2019, 2021; Miller et al. 2017; Siegel and Boine 2019; Siegel et al. 2021; Kravitz-Wirtz et al. 2021a). All panel members who were adults (aged ≥ 18 years) and residents of California were eligible to participate. Most sociodemographic data were collected by Ipsos as part of ongoing KnowledgePanel membership. Data on household and personal firearm ownership (firearm owner, non-owner living with owner, and non-owner in a household without firearms—hereafter, “non-owner”) were collected as part of CSaWS 2020 (see “Methods” in supplement) (Kravitz-Wirtz et al. 2021a).

A final survey weight variable provided by Ipsos adjusted for the initial probability of selection into KnowledgePanel and for survey-specific non-response and over-coverage or under-coverage using poststratification raking ratio adjustments. With weighting, the sample is designed to be statistically representative of the non-institutionalized adult population of California as reflected in the 2014–2018 American Community Survey (ACS).

### Measures

The full text of questions regarding experiences of violence, and the response options, is given in “Methods” in supplement. Participants were asked to report their experiences as of the time of the survey except for encounters with “sidewalk memorials…at places where people died from violence,” which were likely to have been affected by stay-at-home orders related to the COVID-19 pandemic. In that case, participants were asked to base their response on “an average week, before the coronavirus epidemic and staying at home.” Participants were asked to report the number of “people that you personally know,” not including themselves, who “have ever been shot by someone else” and the number who “have ever shot themselves.” A preamble to that section of the questionnaire asked respondents “to think about experiences throughout your entire life.” Those reporting nonzero counts were asked if the shooting(s) had been “an accident” or “on purpose.” Only purposeful shootings were included in this analysis. Knowledge of individuals perceived to be at risk of violence was determined by asking participants if they were concerned that anyone they knew “might physically hurt” another person or themselves “on purpose.” Participants were instructed to “consider only people you know personally, not people you've only heard about from others or seen in the media.”

### Statistical analysis

The analysis was conducted using Stata version 15.1 (StataCorp). Responses for sidewalk memorial encounters and persons known to respondents could range from 0 to 25 and were stratified for analyses. Respondents who reported knowing persons who had been shot or were perceived to be at risk of violence were coded as refusals if they did not answer questions about the intent of the shooting or the number of persons about whom they were reporting.

To generate prevalence estimates for EVs, we calculated weighted percentages and 95% confidence intervals (CIs) for each measure or cross-tabulation of measures using Stata’s survey and weighting commands. Prevalence takes 3 forms here: point prevalence for gunshots and shootings and present knowledge of persons perceived to be at risk of violence, period prevalence for seeing sidewalk memorials “In an average week,” and lifetime prevalence for knowledge of persons who had been shot or shot themselves.

Estimated counts of California adults with each EV were generated by simple extrapolation, multiplying the weighted proportion exposed in our sample by the estimated adult population of California as of the 2018 ACS (30.075 million persons).

Correlation coefficients between EVs were small (Additional file 1: Table S1). We therefore created 4 aggregate measures: (1) the total number of reported EVs; (2) the total number of known persons who had purposefully been shot by others or shot themselves; (3) the total number of known persons who were perceived to be at risk of violence to others or themselves; and (4) the combination of aggregates 2 and 3: the total number of known persons who had purposefully been shot or were perceived to be at risk of violence.

For aggregates 2–4, duplication was possible (for example, a respondent might report the same individual as being at risk of violence to others and to themselves). We generated upper-bound estimates that made no allowance for duplication (estimates based on the sum of known persons reported across all EVs included in the aggregate) and lower-bound estimates that assumed complete duplication (estimates based on the largest number of known persons reported for any 1 of the EVs included in the aggregate).

## Results

Of 5018 panel members invited to participate, 2870 (57%) completed the survey. Most respondents (52.3%, 95% CI 49.5–55.0%) were female; 41.9% (95% CI 39.3–44.6%) were white, 34.7% (95% CI 32.0–37.4%) were Latinx, 14.4% (95% CI 12.3–16.8%) were Asian-American, and 5.8% (95% CI 4.6–7.3%) were Black. Mean [SD] participant age was 47.9 [16.9] years. Firearm owners accounted for 15.2% (95% CI 13.4–17.2%) of respondents, non-owners living with owners for 8.3% (95% CI 6.9–10.0%), and non-owners for 71.4% (95% CI 68.8–73.8%). Non-owners who lived with owners were predominantly female (74.9%; 95% CI 64.9–82.9%), while owners were predominantly male (73.5%; 95% CI 67.6–78.7%). Compared to non-respondents, respondents were slightly more often male and non-Latinx, were older, and had higher income (Additional file 1: Table S2).

Item non-response for EV questions was 0.8% for gunshots and shootings; 4.4% for sidewalk memorials; 5.2% and 3.7%, respectively, for knowing people who were shot by others or shot themselves; and 0.8% and 0.6%, respectively, for knowing people perceived to be at risk of violence to others or themselves. Non-response for firearm ownership was 0.8%.

### Prevalence and extent of experiences of violence

Among all 2870 respondents, 7.4% (95% CI 5.9–9.2%) considered “gunshots and shootings in [their] neighborhood” to be a “big problem” (Table 1). Other EVs were widespread. More than 40% (42.8%, 95% CI 40.1–45.6%) of respondents encountered 1 or more sidewalk memorials in an average week; 1 in 5 (19.7%, 95% CI 17.5–21.9%) personally knew at least 1 person who had been “shot by someone else on purpose”; 1 in 7 (14.6%, 95% CI 13.0–16.5%) knew at least 1 person who had “shot themselves on purpose”; and approximately 1 in 8 knew at least 1 person they perceived to be at risk of violence to others (11.8%, 95% CI 10.1–13.7%) or to themselves (13.0%, 95% CI 11.2–15.0%).

**Table 1:** Prevalence and extent of experiences of violence (EVs) among respondents and estimates for the adult population of California

EV type	Respondents (*n* = 2870)		Estimated N of adults in California
	Unweighted *n*	Weighted % (95% CI)	N (95% CI) (in millions)
*Community environment*			
These days…how much of a problem are gunshots and shootings in your neighborhood?			
Not a problem	1909	62.3 (59.5–65.0)	18.7 (17.9–19.5)
Small problem	557	21.1 (18.8–23.5)	6.3 (5.7–7.1)
Big problem	160	7.4 (5.9–9.2)	2.2 (1.8–2.8)
In an average week…how many sidewalk memorials did you see at places where people died from violence?			
0	1625	52.8 (50.0–55.6)	15.9 (15.0–16.7)
1	514	18.7 (16.6–21.0)	5.6 (5.0–6.3)
2–3	483	17.4 (15.3–19.6)	5.2 (4.6–5.9)
≥ 4	165	6.8 (5.5–8.5)	2.0 (1.7–2.6)
*Social network*			
How many people that you know personally have ever been shot by someone else on purpose?			
0	2146	73.1 (70.5–75.5)	22.0 (21.2–22.7)
1	297	9.5 (8.0–11.2)	2.9 (2.4–3.4)
≥ 2	275	10.1 (8.6–12.0)	3.0 (2.6–3.6)
How many people that you know personally have ever shot themselves on purpose?			
0	2219	80.5 (78.3–82.5)	24.2 (23.5–24.8)
1	411	11.0 (9.6–12.6)	3.3 (2.9–3.8)
≥ 2	136	3.6 (2.7–4.7)	1.1 (0.8–1.4)
Are you concerned that anyone you know might physically hurt another person on purpose?			
No	2528	87.4 (85.4–89.2)	26.3 (25.7–26.8)
Yes, 1 person	195	7.2 (5.8–8.8)	2.2 (1.7–2.6)
Yes, ≥ 2 persons	126	4.6 (3.6–5.9)	1.4 (1.1–1.8)
Are you concerned that anyone you know might physically hurt themselves on purpose?			
No	2469	86.4 (84.4–88.2)	26.0 (25.4–26.5)
Yes, 1 person	226	7.8 (6.4–9.5)	2.3 (1.9–2.9)
Yes, ≥ 2 persons	158	5.1 (4.0–6.4)	1.5 (1.2–1.9)
*Aggregate measures*			
Number of distinct EVs			
0	1000	35.4 (32.7–38.1)	10.6 (9.8–11.5)
1	1013	34.9 (32.3–37.6)	10.5 (9.7–11.3)
2	528	18.4 (16.3–20.6)	5.5 (4.9–6.2)
≥ 3	329	11.4 (9.7–13.2)	3.4 (2.9–4.0)
Number of known persons who have been shot or shot themselves on purpose			
0	1863	67.4 (64.8–69.9)	20.3 (19.5–21.0)
Upper bound^a^			
1	496	14.1 (12.4–15.9)	4.3 (3.7–4.8)
≥ 2	455	15.1 (13.2–17.2)	4.5 (4.0–5.2)
Lower bound^a^			
1	569	16.1 (14.4–18.1)	4.8 (4.3–5.4)
≥ 2	382	13.0 (11.3–15.0)	3.9 (3.4–4.5)
Number of known persons perceived to be at risk of violence			
0	2255	78.5 (76.1–80.7)	23.6 (22.9–24.3)
Upper bound^a^			
1	317	11.1 (9.5–13.0)	3.4 (2.9–3.9)
≥ 2	287	9.9 (8.4–11.7)	3.0 (2.5–3.5)
Lower bound^a^			
1	351	12.5 (10.7–14.5)	4.8 (3.2–4.4)
≥ 2	253	8.5 (7.1–10.2)	2. 6 (2.1–3.1)
Combined social network EVs: number of known persons who have been shot or shot themselves on purpose or are perceived to be at risk of violence			
0	1600	58.8 (56.1–61.5)	17.7 (16.8–18.5)
Upper bound^a^			
1	534	16.5 (14.6–18.6)	5.0 (4.4–5.6)
≥ 2	731	24.4 (22.1–26.8)	7.3 (6.6–8.1)
Lower bound^a^			
1	687	21.2 (19.1–23.5)	6.4 (5.7–7.1)
≥ 2	578	19.7 (17.6–22.0)	5.9 (5.3–6.6)

Refusals and “don’t know” responses are not shown. See “Methods” in supplement for exact text of questions

^a^See “Methods” section for details of calculation

Nearly two-thirds of respondents (64.6%, 95% CI 61.9–67.3%) reported at least 1 EV, and 11.4% (95% CI 9.7–13.2%) reported 3 or more (Table 1). Based on upper-bound estimates, 1 in 6 respondents (15.1%; 95% CI 13.2%-17.2%) personally knew 2 or more people who had purposefully been shot, and 1 in 10 (9.9%, 95% CI 8.4%, 11.7%) knew 2 or more people they perceived to be at risk of violence. More than 40% of respondents (40.9%; 95% CI 38.2%, 43.6%) knew at least 1 person who had purposefully been shot or was perceived to be at risk of violence.

Extrapolations to the statewide adult population (Table 1) suggest that each EV would be reported by many California adults. Experience would often be extensive, with an estimated 3.4 million Californians having 3 or more EVs, 4.5 million knowing 2 or more people who have purposefully been shot, and 3.0 million knowing 2 or more people whom they perceive to be at risk of violence.

### Associations with respondent characteristics

There were two consistent findings (Tables 2, 3; Additional file 1: Tables S3, S4). Gender was not associated with the prevalence of any EV. Second, when respondents were stratified by firearm ownership, non-owners who lived with owners reported both a higher prevalence and greater extent of social network EVs than did firearm owners or non-owners (Table 3). For example, 11.2% (95% CI 6.0–19.8%) of non-owners who lived with owners, but only 3.4% (95% CI 1.9–5.8%) of firearm owners and 3.9% (95% CI 2.9–5.2%) of non-owners, knew 2 or more persons they perceived to be at risk of violence to others.

**Table 2:** Prevalence and extent of community environment experiences of violence by respondents’ age, race/ethnicity, gender, and firearm ownership (*n* = 2870)

Respondent characteristic	These days…how much of a problem are gunshots and shootings in your neighborhood? Weighted row % (95% CI)			In an average week…how many sidewalk memorials did you see at places where people died from violence? Weighted row % (95% CI)		
	Not a problem	Small problem	Big problem	0	1	≥ 2
*Age*						
18–29	62.1 (53.7–69.8)	21.5 (15.5–29.1)	7.9 (4.4–13.7)	49.8 (41.5–58.2)	17.0 (11.7–24.1)	27.4 (20.6–35.4)
30–44	52.4 (46.6–58.2)	25.8 (21.0–31.4)	10.1 (6.9–14.4)	47.4 (41.7–53.2)	20.2 (15.8–25.4)	26.4 (21.5–31.8)
45–59	61.2 (56.0–66.1)	22.8 (18.6–27.5)	7.3 (4.9–10.8)	51.5 (46.2–56.6)	19.7 (15.9–24.2)	24.3 (20.3–28.7)
60 +	74.3 (70.8–77.5)	14.0 (11.7–16.5)	4.2 (2.6–6.7)	61.7 (58.0–65.2)	16.9 (14.4–19.8)	19.8 (16.9–23.1)
*Race/ethnicity*						
White	72.3 (68.8–75.6)	16.2 (13.6–19.1)	3.7 (2.3–5.8)	61.8 (58.1–65.4)	16.3 (13.9–19.0)	19.7 (16.7–23.1)
Black	60.4 (48.2–71.5)	18.4 (10.8–29.6)	12.8 (6.0–25.2)	38.5 (27.4–51.0)	24.3 (15.3–36.3)	30.7 (21.3–42.1)
Asian-American	66.1 (57.3–73.9)	18.6 (12.6–26.5)	7.2 (3.7–13.6)	58.9 (50.3–67.0)	22.3 (16.0–30.3)	15.2 (10.1–22.3)
Multiracial or other	75.9 (59.0–87.3)	9.5 (3.9–21.5)	7.3 (1.4–30.1)	62.4 (45.7–76.6)	12.0 (5.6–24.1)	25.6 (14.0–42.1)
Latinx	47.6 (42.7–52.6)	29.5 (25.0–34.4)	11.0 (8.3–14.6)	40.8 (36.1–45.7)	19.7 (15.8–24.2)	32.1 (27.7–36.9)
*Gender*						
Male	61.1 (57.0–65.1)	23.4 (20.0–27.2)	7.2 (5.2–10.0)	54.2 (50.0–58.2)	17.9 (14.9–21.2)	23.2 (19.9–26.9)
Female	63.4 (59.6–67.0)	18.9 (16.1–22.2)	7.5 (5.6–10.0)	51.6 (47.7–55.4)	19.4 (16.5–22.7)	25.1 (21.9–28.6)
*Household firearm ownership*						
Non-owner	60.1 (56.8–63.4)	22.0 (19.2–24.9)	8.2 (6.4–10.4)	51.2 (47.9–54.5)	19.2 (16.7–22.0)	25.3 (22.5–28.4)
Firearm owner	71.6 (64.7–77.6)	19.0 (14.0–25.2)	3.3 (1.2–8.7)	60.4 (53.5–66.9)	15.7 (11.6–20.8)	20.2 (15.0–26.6)
Non-owner living with owner	64.4 (54.4–73.3)	18.1 (11.9–26.4)	10.8 (5.4–20.5)	54.5 (44.7–63.9)	19.0 (12.6–27.5)	26.2 (18.8–35.4)

Refusals and “don’t know” responses are not shown. See “Methods” in supplement for exact text of questions

**Table 3:** Prevalence and extent of social network experiences of violence by respondents’ age, race/ethnicity, gender, and firearm ownership (*n* = 2870)

Respondent characteristic	How many people that you know personally have ever been shot by someone else on purpose? Weighted row % (95% CI)			How many people that you know personally have ever shot themselves on purpose? Weighted row % (95% CI)			How many people that you know might physically hurt another person on purpose? Weighted row % (95% CI)			How many people that you know might physically hurt themselves on purpose? Weighted row % (95% CI)		
	0	1	≥ 2	0	1	≥ 2	0	1	≥ 2	0	1	≥ 2
*Age*												
18–29	73.0 (65.0–79.7)	8.4 (4.9–14.2)	12.5 (7.8–19.4)	85.3 (79.1–89.9)	7.6 (4.7–11.9)	3.2 (1.1–8.6)	84.7 (77.5–89.9)	8.1 (4.5–14.2)	6.3 (3.2–12.2)	76.8 (68.8–83.2)	11.9 (7.2–19.0)	11.1 (7.0–17.2)
30–44	70.3 (64.7–75.3)	7.5 (5.1–10.9)	10.8 (7.7–14.9)	80.2 (75.3–84.4)	8.0 (5.7–11.0)	3.4 (1.9–5.9)	84.9 (80.6–88.5)	8.8 (6.0–12.6)	5.2 (3.4–7.8)	86.5 (82.5–89.7)	6.7 (4.6–9.7)	5.2 (3.4–8.1)
45–59	68.3 (63.2–73.0)	12.1 (9.0–16.2)	12.5 (9.6–16.1)	80.1 (75.7–83.8)	11.8 (9.0–15.3)	3.9 (2.4–6.3)	87.1 (83.2–90.2)	7.8 (5.3–11.4)	4.0 (2.6–6.2)	87.8 (83.9–90.8)	8.2 (5.6–11.9)	3.7 (2.4–5.8)
60 +	80.8 (77.9–83.4)	9.7 (7.9–12.0)	5.7 (4.3–7.6)	78.2 (75.2–81.0)	15.7 (13.3–18.4)	3.8 (2.8–5.2)	92.0 (90.0–93.6)	4.2 (3.2–5.6)	3.4 (2.3–5.1)	90.7 (88.8–92.3)	6.3 (4.9–8.0)	2.8 (2.0–3.9)
*Race/ethnicity*												
White	80.5 (77.3–83.4)	8.7 (7.1–10.8)	7.0 (5.1–9.6)	76.1 (73.0–79.0)	16.2 (13.9–18.7)	5.0 (3.8–6.6)	88.5 (86.0–90.6)	6.6 (5.1–8.6)	4.2 (2.9–5.9)	83.1 (80.0–85.9)	9.6 (7.6–12.2)	6.7 (5.0–8.9)
Black	49.3 (37.5–61.2)	12.1 (6.6–21.1)	31.0 (20.9–43.3)	83.0 (72.5–90.1)	7.6 (3.6–15.5)	2.6 (0.9–7.4)	90.6 (82.9–95.0)	4.8 (1.8–12.1)	3.5 (1.5–8.1)	93.1 (81.6–97.6)	5.9 (1.7–18.2)	1.0 (0.25–4.1)
Asian-American	84.0 (76.9–89.2)	9.2 (5.3–15.4)	3.7 (1.6–8.7)	90.0 (83.6–93.8)	6.1 (3.3–11.1)	1.4 (0.2–9.4)	89.2 (81.8–93.8)	9.9 (5.5–17.3)	0.25 (0.04–1.8)	89.6 (82.8–94.0)	8.5 (4.6–15.4)	1.6 (0.5–4.8)
Multiracial or other	74.1 (55.2–86.9)	10.9 (3.4–29.7)	14.0 (5.2–32.3)	78.7 (61.3–89.6)	9.5 (3.9–21.3)	7.6 (1.5–30.5)	81.9 (62.2–92.6)	6.0 (1.1–26.0)	12.1 (4.0–31.4)	76.6 (57.8–88.7)	14.2 (5.6–31.3)	9.2 (2.5–28.9)
Latinx	63.4 (58.4–68.1)	10.0 (7.4–13.5)	12.7 (9.9–16.1)	81.6 (77.4–85.3)	7.6 (5.6–10.3)	2.6 (1.4–4.7)	85.3 (81.5–88.5)	7.2 (4.9–10.4)	6.4 (4.4–9.1)	88.8 (85.5–91.4)	5.1 (3.5–7.6)	5.0 (3.3–7.5)
*Gender*												
Male	73.4 (69.6–76.8)	7.8 (6.0–10.0)	11.5 (9.1–14.3)	80.9 (77.7–83.8)	9.8 (8.0–12.0)	3.4 (2.4–4.8	87.1 (84.1–89.6)	8.0 (5.9–10.7)	3.8 (2.7–5.3)	87.6 (84.6–90.1)	7.1 (5.2–9.6)	4.1 (2.8–6.0)
Female	72.8 (69.1–76.1)	11.1 (8.9–13.7)	8.9 (6.9–11.4)	80.0 (77.0–82.8)	12.1 (10.1–14.4)	3.8 (2.5–5.7)	87.7 (84.9–90.0)	6.4 (4.7–8.6)	5.3 (3.8–7.4)	85.3 (82.4–87.8)	8.5 (6.6–10.9)	6.0 (4.5–8.0)
*Household firearm ownership*												
Non-owner	75.2 (72.2–78.0)	8.2 (6.7–10.1)	8.5 (6.8–10.5)	81.9 (79.3–84.2)	10.2 (8.5–12.0)	2.9 (2.0–4.2)	88.7 (86.4–90.6)	6.9 (5.3–8.8)	3.9 (2.9–5.2)	87.0 (84.7–89.1)	8.1 (6.4–10.1)	4.5 (3.5–5.9)
Firearm owner	69.6 (63.0–75.5)	13.1 (9.4–17.9)	13.9 (9.7–19.3)	77.8 (72.4–82.4)	14.2 (10.8–18.4)	5.5 (3.5–8.5)	88.7 (84.0–92.1)	7.4 (4.5–11.9)	3.4 (1.9–5.8)	88.7 (83.8–92.3)	7.1 (4.5–10.9)	4.2 (2.0–8.6)
Non-owner living with owner	65.7 (55.6–74.7)	15.0 (8.7–24.6)	16.4 (10.1–25.7)	77.1 (68.9–83.6)	12.8 (8.5–18.6)	7.9 (3.8–15.7)	78.7 (68.8–86.1)	9.8 (5.0–18.4)	11.2 (6.0–19.8)	76.8 (67.0–84.3)	9.0 (4.7–16.7)	13.6 (7.8–22.7)

Refusals and “don’t know” responses are not shown. See “Methods” in supplement for exact text of questions

Other associations varied with the EV and the respondent characteristic (Tables 2, 3, Additional file 1: Tables S3, S4). The 2 community environment EVs, for example, were more common among Latinx and Black respondents; inversely associated with age, income and education; and unrelated to marital status (Table 2; Additional file 1: Table S3).

Knowledge of people who had been shot by others was least common among respondents aged ≥ 60 years, but knowledge of people who had shot themselves was most common in that group (Table 3). Knowledge of people who had been shot by others was much more prevalent and extensive among Black respondents, 31.0% (95% CI 20.9–43.3%) of whom knew 2 or more such persons, than among others (Table 3). Knowledge of people who had shot themselves was most common among white respondents, but differences among racial/ethnic groups were much less pronounced. Social network exposures were generally most common among never-married or widowed respondents and bore variable relationships to education and income (Additional file 1: Table S4).

Knowledge of people perceived to be at risk of violence did not follow these patterns (Table 3) and did not vary substantially with respondents’ race/ethnicity. Knowledge of people perceived to be at risk of violence to themselves was highest among respondents aged 18–29 and decreased steadily thereafter.

Reports of multiple EVs were least common among respondents aged ≥ 60 years, were unrelated to gender, and were most frequent among Black, Other/multiracial, and Latinx respondents and among non-owners of firearms who lived with owners (Table 4). There was little variation in relation to marital status, education, or income (Additional file 1: Table S5).

**Table 4:** Total number of experiences of violence (EVs) by respondents’ age, race/ethnicity, gender, and firearm ownership

Respondent characteristic	Total number of reported EVs Weighted % (95% CI)			
	0	1	2	≥ 3
*Age*				
18–29	25.1 (18.5–33.1)	42.4 (34.3–51.0)	22.9 (16.5–30.7)	9.6 (5.9–15.2)
30–44	35.5 (30.0–41.3)	34.0 (28.7–39.8)	17.8 (13.8–22.5)	12.8 (9.5–17.0)
45–59	36.4 (31.5–41.5)	31.2 (26.9–35.8)	17.3 (13.8–21.4)	15.2 (11.7–19.5)
60+	40.4 (36.7–44.1)	35.1 (31.7–38.7)	17.4 (14.7–20.5)	7.1 (5.6–9.1)
*Race/ethnicity*				
White	35.9 (32.4–39.5)	37.0 (33.4–40.7)	17.8 (15.2–20.8)	9.3 (7.5–11.6)
Black	26.5 (17.1–38.7)	26.7 (17.6–38.2)	33.4 (22.7–46.2)	13.5 (8.0–21.7)
Asian-American	48.9 (40.5–57.4)	31.9 (24.7–40.1)	9.8 (5.9–15.9)	9.4 (5.2–16.5)
Multiracial or other	26.2 (14.1–43.4)	41.4 (25.9–58.8)	18.5 (8.4–36.1)	14.0 (5.3–32.0)
Latinx	31.4 (26.9–36.2)	34.5 (29.9–39.4)	20.1 (16.3–24.4)	14.1 (11.0–17.8)
*Gender*				
Male	38.4 (34.4–42.5)	33.5 (29.8–37.4)	17.0 (14.2–20.3)	11.2 (8.8–14.0)
Female	32.6 (29.2–36.3)	36.2 (32.6–40.0)	19.6 (16.7–22.9)	11.6 (9.4–14.2)
*Household firearm ownership*				
Non-owner	36.0 (32.8–39.2)	35.9 (32.8–39.2)	17.4 (15.0–20.0)	10.8 (8.9–13.0)
Firearm owner	38.8 (32.2–45.8)	30.4 (24.9–36.6)	18.0 (13.6–23.6)	12.7 (8.8–18.1)
Non-owner living with owner	18.4 (12.6–26.2)	36.8 (28.1–46.5)	29.2 (20.5–39.7)	15.6 (9.9–23.7)

Findings for aggregated social network EVs were similar (Additional file 1: Tables S6–S9). Knowledge of 2 or more persons who had purposefully been shot or were at risk of violence (Additional file 1: Table S8) was reported by 30.9% (95% CI 23.7–39.1%) of respondents aged 18–29 years, 35.7% (95% CI 25.2–47.8%) of Black respondents, and 41.3% (95% CI 31.8–51.4%) of non-owners of firearms who lived with owners.

## Discussion

The goal of this survey study was to examine a broad range of experiences of violence in daily life in an adult general population sample. Such experiences are widespread, affecting nearly two-thirds of respondents. In every subgroup examined, 50–75% of respondents reported at least 1 of the 6 EVs; in most cases, 10–15% reported 3 or more. Statewide in California, an estimated 8.9 million adults personally know at least 1 person who has been shot or shot themselves on purpose and 6.3 million know someone whom they perceive to be at risk of violence to themselves or others.

Two consistent findings are particularly noteworthy. Respondents’ gender was not associated with any EV, a striking contrast with the much-increased risk among men for most forms of violent victimization. Studies limited to victimization underestimate the impact of violence on women.

Second, non-owners of firearms who lived with owners reported more prevalent and extensive social network EVs than others did. They were much more likely than firearm owners and non-owners in households without firearms to know persons they perceived to be at risk of violence.

The EV burdens of what might be termed “secondhand firearm ownership” in this group deserve further study that addresses the following questions, among others: As three-fourths of non-owners who lived with owners were women, how much does the prevalence and extent of EVs in this group account for the overall lack of association between EVs and gender? What are the consequences of these EVs? Do the persons these respondents perceive to be at risk of violence own firearms? Are they the partners or household members of the respondents? Where the perceived risk is for violence to others, are the respondents potential targets? What firearm violence prevention interventions might enlist non-owners who live with owners to help identify persons at risk or deliver the interventions? Extreme risk protection orders (ERPOs) are a potential example; in another study of this population, non-owners who lived with owners reported the highest levels of willingness to request an ERPO for a family member (Kravitz-Wirtz et al. 2021).

Other specific findings deserve comment. Black and Latinx respondents were much more likely than others to report EVs related to the community environment and interpersonal violence; white respondents were more likely to report EVs related to self-directed violence. Knowledge of people who had shot themselves increased steadily with respondents’ age, a finding consistent with the relationship between age and suicide risk among men, but knowledge of people who were perceived to be at risk of violence to themselves was most common among respondents aged 18–29 years and decreased thereafter. It might be that the first finding reflects an age effect and knowledge accumulated over time, while the latter reflects current information and younger adults’ larger social networks (Bruin et al. 2020). No subset of the population was free of EVs, and aggregate measures tended to show smaller variation with respondent characteristics than individual experiences did.

In high-risk populations, experiences like those we examined are extremely common. Hearing gunshots, for example, has been reported by 73% of adult residents (*n* = 1189) of low-income neighborhoods in New Haven, Connecticut (Santilli et al. 2017); 74% of 107 households with children in “a highly impoverished and racially/ethnically segregated city” in the Middle Atlantic region (Echeverria et al. 2014); and 84% of 31 mothers of children participating in Head Start in Southern California (Aisenberg 2001). Among 1615 adults in 4 large East Coast cities, 24% knew someone who “died due to gun violence” (Smith et al. 2020). In the New Haven study, 16% of respondents had been present at a shooting scene, and 69% of them knew the victims (Santilli et al. 2017).

We did not identify peer-reviewed studies of social network exposures relating to interpersonal violence in the general population, but 2 relevant public opinion polls exist. In 2013, 20% of US adults reported that they “personally know someone who has been the victim of a crime involving a gun in the past 3 years” (Kaiser Health Tracking Poll 2013). In 2017, 44% reported “that someone they know has been shot, either accidentally or intentionally” (Parker et al. 2017). One peer-reviewed study of General Social Survey data for self-directed violence found that 51% of respondents knew 1 or more persons who had died by suicide” (Feigelman et al. 2018). Given differences in the measures examined, these findings are not directly comparable to ours, but they confirm that such experiences of violence are common.

### Limitations

This study has the limitations inherent in survey research. The findings are cross-sectional and subject to sampling error, bias due to non-response and other factors, and unmeasured confounding. No claims of causal relationships can be made based on the associations we found. Other limitations are specific to this study. Item non-response exceeded 3% for 3 exposures. Recall error may be higher than usual given the stresses associated with the COVID-19 pandemic. EV questions were not validated. We did not measure acute victimization, whether direct or indirect (doing so would have increased the time required to take the survey and reduced the response rate) and are not able to assess relationships between victimization and the experiences we studied. We did not prohibit respondents from listing a person known to them in multiple social network EVs, but our upper- and lower-bound estimates for EVs are very similar. Our aggregate measures could be criticized as combining multiple measurements of similar experiences, but correlation coefficients were small. Even if they were not, the aggregates would remain appropriate measures of the extent of experiences of violence.

Two limitations on generalizability deserve comment. The survey was conducted during the COVID-19 pandemic, and findings for some EVs, such as knowledge of persons perceived to be at risk of violence, may not hold outside the unique circumstances of the pandemic period. Second, these are single-state findings; grim as they are, they almost certainly underestimate the situation in most other states, particularly for self-directed violence. In 2019, California placed 29th among the 50 states ranked from high to low on their age-adjusted firearm homicide rates and 46th of 50 on firearm suicide (Centers for Disease Control and Prevention 2021). We recommend that comparable surveys be performed in other states.

## Conclusion

Experiences of violence in daily life are widespread in the general adult population; many occur in clear patterns that are unlike those seen for violent victimization. As with victimization, it is plausible that these experiences have durable and cumulative adverse consequences. Further study of the prevalence and extent of these and other experiences of violence, and their effects, will help develop a more comprehensive understanding of violence and the burdens it imposes on the general population. Such an understanding would assist the development and evaluation of violence prevention interventions.

## Supplementary Information

**Additional file 1.** SUPPLEMENT: Experiences of Violence in Daily Life among Adults in California: A Population-Representative Survey.

## Data Availability

Data for this study will be made available when CSaWS 2020 analyses are complete.
