# Supplementary material for: Experiences of violence in daily life among adults in California: a population-representative survey
Source: Inj Epidemiol. 2022 Jan 3;9:1. doi: 10.1186/s40621-021-00367-1 (PMC8721630; doi:10.1186/s40621-021-00367-1)
Supplement: Supplementary file 1 — Additional file 1. SUPPLEMENT: Experiences of Violence in Daily Life among Adults in California: A Population-Representative Survey. [file 40621_2021_367_MOESM1_ESM.docx]

**SUPPLEMENT**

**Experiences of Violence in Daily Life among Adults in California: A Population-Representative Survey**

Garen J. Wintemute, MD, MPH; Amanda J. Aubel, MPH; Rocco Pallin, MPH; Julia P. Schleimer, MPH; Nicole Kravitz-Wirtz, PhD, MPH

**Contents**

Methods. Selected survey items and response options: p 2

Supplemental Table 1. Correlation matrix for experience of violence variables: p 5

Supplemental Table 2. Characteristics of respondents and non-respondents: p 6

Supplemental Table 3. Prevalence and extent of community environment experiences of violence by respondents’ marital status, education, and income: p 7

Supplemental Table 4. Prevalence and extent of social network experiences of violence by respondents’ marital status, education, and income: p 8

Supplemental Table 5. Total number of experiences of violence by respondents’ marital status, education, and income: p 10

Supplemental Table 6. Combined social network experiences of violence by respondents’ age, race/ethnicity, gender, and firearm ownership: p 11

Supplemental Table 7. Combined social network experiences of violence by respondents’ marital status, education, and income: p 13

Supplemental Table 8. Total social network experiences of violence by respondents’ age, race/ethnicity, gender, and firearm ownership: p 15

Supplemental Table 9. Total social network experiences of violence by respondents’ marital status, education, and income: p 16

Methods. Selected survey items and response options

**Determining Firearm Ownership**

Now we have some questions about guns. In this survey, we use the word gun to refer to any firearm, including pistols, revolvers, shotguns, and rifles. This does not include air guns, BB guns, starter pistols, or paintball guns.

**Q104.** Do you or does anyone else you live with currently own any type of gun?

*Responses:*

1. Yes

2. No

3. Don’t know

[Asked if Q104=1]

**Q105.** Do you personally own a gun?

*Responses:*

1. Yes

2. No

**Experience 1. These days, during the coronavirus epidemic, how much of a problem are gunshots and shootings in your neighborhood?**

Now we’re going to ask you some questions about your neighborhood, before and during the coronavirus epidemic.

**Q19.** These days, during the coronavirus epidemic, how much of a problem are gunshots and shootings in your neighborhood?

*Responses:*

1. Big problem

2. Small problem

3. Not a problem

4. Don’t know

**Experience 2. In an average week, before the coronavirus epidemic and staying at home, how many sidewalk memorials did you see at places where people died from violence?**

Next we’ll ask about “sidewalk memorials,” where people sometimes put candles, flowers, and other items at places where people have died.

**Q24.** In an average week, before the coronavirus epidemic and staying at home, how many sidewalk memorials did you see at places where people died from violence? If you’re not sure, please make your best guess.

*Responses:*

[Dropdown with options 0-25, and 26 or more]

**Experience 3.** **How many people that you know personally have ever been shot by someone else?**

Now we’d like you to think about experiences throughout your entire life, not just before your 18th birthday.

**Q27.** Not counting yourself, how many people that you personally know have ever been shot by someone else? Do not count people in the armed forces who were shot in the line of duty.

*Responses:*

[Dropdown with options 0-25]

**Q28.** Was this an accident or on purpose? (For response= 1 to **Q27**.)

1. An accident

2. On purpose

3. Don’t know

**Q29.** You said you personally know [insert number from **Q27**] people who have been shot by someone else. How many of these people were shot by accident? (For response> 1 to **Q27**.)

*Responses:*

[Dropdown with options 0-25]

**Q30.** How many of these people were shot on purpose? (For response> 1 to **Q27**.)

*Responses:*

[Dropdown with options 0-25]

**Experience 4.** **How many people that you know personally have ever shot themselves?**

**Q31.** Not counting yourself, how many people that you personally know have ever shot themselves?

*Responses:*

[Dropdown with options 0-25]

**Q32.** Was this an accident or on purpose? (For response= 1 to **Q31**.)

*Responses:*

1. An accident

2. On purpose

3. Don’t know

**Q33.** You said you personally know [insert number from **Q31**] people who have shot themselves. How many of these people shot themselves by accident? (For response> 1 to **Q31**.)

*Responses:*

[Dropdown with options 0-25]

**Q34.** How many of these people shot themselves on purpose? (For response> 1 to **Q31**.)

*Responses:*

[Dropdown with options 0-25]

**Experience 5. Are you concerned that anyone you know might physically hurt another person on purpose?**

**Q35.** Are you concerned that anyone you know might physically hurt another person on purpose? Consider only people you know personally, not people you've only heard about from others or seen in the media.

*Responses:*

1. Yes

2. No

**Q36.** How many of these people do you know? (If response = “Yes” to **Q35**.)

*Responses:*

[Dropdown with options 0-25]

**Experience 6.** **Are you concerned that anyone you know might physically hurt themselves on purpose?**

**Q63.** Are you concerned that anyone you know might physically hurt themselves on purpose? Consider only people you know personally, not people you've only heard about from others or seen in the media.

*Responses:*

1. Yes

2. No

**Q64.** How many of these people do you know? (If response = “Yes” to **Q63**.)

*Responses:*

[Dropdown with options 0-25]

**Supplemental Table 1. Correlation matrix for experience of violence variables**

|  | **Gunshots** | **Memorials** | **Shot by others** | **Shot self** | **Risk to others** | **Risk to self** |
| --- | --- | --- | --- | --- | --- | --- |
| Gunshots | 1.0000 |  |  |  |  |  |
| Memorials | 0.1934 | 1.0000 |  |  |  |  |
| Shot by others | 0.1434 | 0.1611 | 1.0000 |  |  |  |
| Shot self | -0.0226 | -0.0154 | 0.1379 | 1.0000 |  |  |
| Risk to others | 0.1123 | 0.0925 | 0.1309 | 0.0643 | 1.0000 |  |
| Risk to self | 0.0733 | 0.0486 | 0.0907 | 0.1140 | 0.1780 | 1.0000 |

**Supplemental Table 2. Characteristics of respondents and non-respondents^a^**

| **Characteristic** | **Respondents (n= 2870)** | | **Non-respondents**  **(n= 2148)** | |
| --- | --- | --- | --- | --- |
|  | **Unweighted n** | **Weighted % (95% CI)** | **n** | **%** |
| Age |  |  |  |  |
| 18-29 | 200 | 16.3 (14.0-18.9) | 496 | 23.1 |
| 30-44 | 511 | 29.7 (27.0-32.5) | 586 | 27.3 |
| 45-59 | 738 | 26.7 (24.4-29.2) | 604 | 28.1 |
| 60+ | 1421 | 27.3 (25.3-29.4) | 462 | 21.5 |
| Race/ethnicity |  |  |  |  |
| White | 1615 | 41.9 (39.3-44.6) | 715 | 33.3 |
| Black | 127 | 5.8 (4.6-7.3) | 107 | 5.0 |
| Other | 220 | 15.5 (13.4-18.0) | 140 | 6.5 |
| Multiracial | 59 | 2.1 (1.4-3.1) | 38 | 1.8 |
| Latinx | 849 | 34.7 (32.0-37.4) | 1148 | 53.5 |
| Gender |  |  |  |  |
| Male | 1506 | 47.7 (45.0-50.5) | 925 | 43.1 |
| Female | 1364 | 52.3 (49.5-55.0) | 1223 | 56.9 |
| Marital status |  |  |  |  |
| Married | 1704 | 61.0 (58.2-63.7) | 1216 | 56.6 |
| Widowed | 204 | 4.4 (3.6-5.3) | 93 | 4.3 |
| Divorced | 432 | 11.1 (9.6-12.8) | 288 | 13.4 |
| Never married | 530 | 23.5 (21.1-26.1) | 551 | 25.7 |
| Education |  |  |  |  |
| Less than high school | 158 | 15.4 (13.1-18.0) | 234 | 10.9 |
| High school | 364 | 20.4 (18.1-23.0) | 377 | 17.6 |
| Some college | 938 | 32.7 (30.2-35.2) | 755 | 35.2 |
| Bachelor's degree or higher | 1410 | 31.5 (29.1-33.9) | 782 | 36.4 |
| Income |  |  |  |  |
| Less than $25,000 | 431 | 15.1 (13.2-17.2) | 502 | 23.4 |
| $25,000-$59,999 | 744 | 23.9 (21.7-26.3) | 500 | 23.3 |
| $60,000-$99,999 | 757 | 22.6 (20.4-24.9) | 650 | 30.3 |
| $100,000 or more | 938 | 38.5 (35.8-41.3) | 496 | 23.1 |
| Household firearm ownership |  |  |  |  |
| Non-owner | 1989 | 71.4 (68.8-73.8) | — | — |
| Firearm owner | 529 | 15.2 (13.4-17.2) | — | — |
| Non-owner living with owner | 219 | 8.3 (6.9-10.0) | — | — |

^a^This table is expanded from eAppendix 1 in the original report of findings from the 2020 iteration of the California Safety and Wellbeing Survey: Kravitz-Wirtz N, Aubel A, Schleimer J, Pallin R, Wintemute G. Public concern about violence, firearms, and the COVID-19 pandemic in California. JAMA Netw Open. 2021 Jan 4;4(1):e2033484.

**Supplemental Table 3.** **Prevalence and extent of community environment experiences of violence by respondents’** **marital status, education, and income^a,b^ (n=2870)**

| **Respondent Characteristic** | **These days…how much of a problem are gunshots and shootings in your neighborhood?**  **Weighted row % (95% CI)** | | | **In an average week…how many sidewalk memorials did you see at places where people died from violence?**  **Weighted row % (95% CI)** | | |
| --- | --- | --- | --- | --- | --- | --- |
|  | **Not a problem** | **Small problem** | **Big problem** | **0** | **1** | **≥2** |
|  |  |  |  |  |  |  |
| Marital status |  |  |  |  |  |  |
| Married | 62.8 (59.2-66.3) | 22.2 (19.2-25.5) | 5.8 (4.2-7.9) | 54.3 (50.7-57.9) | 17.5 (14.9-20.5) | 23.4 (20.5-26.6) |
| Widowed | 71.3 (61.4-79.6) | 15.6 (9.6-24.3) | 2.9 (1.3-6.4) | 58.3 (48.2-67.7) | 20.8 (13.5-30.7) | 19.0 (12.3-28.3) |
| Divorced | 61.9 (54.5-68.8) | 18.6 (13.4-25.3) | 8.9 (5.2-14.7) | 55.1 (47.8-62.2) | 13.8 (9.9-18.8) | 26.4 (20.3-33.6) |
| Never married | 59.4 (53.1-65.4) | 20.4 (15.9-25.8) | 11.7 (8.0-16.9) | 46.7 (40.5-53.0) | 23.5 (18.5-29.3) | 26.1 (20.9-32.0) |
| Education |  |  |  |  |  |  |
| Less than high school | 42.2 (33.8-51.2) | 29.8 (22.2-38.6) | 13.4 (8.3-21.0) | 38.6 (30.4-47.4) | 19.9 (13.7-27.9) | 33.4 (25.5-42.5) |
| High school | 56.7 (49.8-63.3) | 23.4 (18.0-29.7) | 9.9 (6.5-14.7) | 45.7 (39.0-52.6) | 17.4 (12.8-23.3) | 30.5 (24.7-37.1) |
| Some college | 67.0 (62.7-71.0) | 19.2 (15.9-22.9) | 6.5 (4.4-9.5) | 54.1 (49.6-58.5) | 19.1 (15.7-23.0) | 24.2 (20.7-28.2) |
| Bachelor's degree | 68.9 (63.4-74.0) | 19.0 (14.9-24.0) | 4.0 (2.2-7.3) | 58.5 (52.9-63.8) | 19.9 (15.8-24.8) | 18.5 (14.7-23.2) |
| Advanced degree | 73.4 (67.1-79.0) | 15.1 (10.9-20.6) | 3.3 (1.6-6.8) | 68.8 (62.7-74.2) | 16.6 (12.3-21.9) | 11.6 (8.9-15.0) |
| Income |  |  |  |  |  |  |
| Less than $25,000 | 45.9 (38.9-53.0) | 25.7 (19.6-32.9) | 11.9 (7.7-17.8) | 36.2 (29.9-43.1) | 20.4 (15.4-26.6) | 33.3 (26.7-40.5) |
| $25,000-$59,999 | 56.6 (51.1-61.9) | 23.4 (19.0-28.6) | 10.1 (7.3-13.9) | 45.6 (40.3-51.0) | 17.5 (13.8-22.0) | 33.2 (28.2-38.6) |
| $60,000-$99,999 | 62.1 (56.3-67.5) | 22.4 (18.0-27.6) | 5.6 (3.3-9.4) | 55.2 (49.5-60.7) | 21.7 (17.2-27.0) | 20.9 (16.5-26.1) |
| $100,000 or more | 72.4 (67.7-76.6) | 17.0 (13.6-21.0) | 5.0 (2.9-8.3) | 62.4 (57.6-66.9) | 16.9 (13.6-20.8) | 17.0 (13.7-20.7) |

^a^Refusals and “don’t know” responses are not shown.

^b^ See “Methods” in supplement for exact text of questions.

**Supplemental Table 4.** **Prevalence and extent of social network experiences of violence by respondents’ marital status, education, and income^a,b^ (n=2870)**

| **Respondent Characteristic** | **How many people that you know personally have ever been shot by someone else on purpose?**  **Weighted row % (95% CI)** | | | **How many people that you know personally have ever shot themselves on purpose?**  **Weighted row % (95% CI)** | | | **How many people that you know might physically hurt another person on purpose?**  **Weighted row % (95% CI)** | | | **How many people that you know might physically hurt themselves on purpose?**  **Weighted row % (95% CI)** | | |
| --- | --- | --- | --- | --- | --- | --- | --- | --- | --- | --- | --- | --- |
|  | **0** | **1** | **≥2** | **0** | **1** | **≥2** | **0** | **1** | **≥2** | **0** | **1** | **≥2** |
| Marital status |  |  |  |  |  |  |  |  |  |  |  |  |
| Married | 75.2 (72.0-78.2) | 8.4 (6.6-10.5) | 9.0 (7.2-11.3) | 79.3 (76.3-82.0) | 11.6 (9.8-13.8) | 4.0 (2.8-5.7) | 88.3 (85.9-90.4) | 7.1 (5.4-9.3) | 3.9 (2.8-5.2) | 88.9 (86.7-90.8) | 6.8 (5.2-8.7) | 3.9 (2.9-5.2) |
| Widowed | 78.7 (69.5-85.8) | 16.0 (9.7-25.4) | 4.1 (1.9-8.6) | 75.8 (66.2-83.4) | 13.8 (8.4-21.7) | 6.8 (2.9-15.2) | 93.5 (87.4-96.7) | 1.7 (0.7-3.8) | 3.2 (0.9-10.3) | 88.5 (80.9-93.3) | 7.4 (3.9-13.8) | 3.4 (1.1-10.4) |
| Divorced | 68.9 (61.5-75.4) | 12.6 (8.3-18.6) | 11.5 (7.3-17.7) | 78.2 (72.6-82.8) | 13.2 (9.7-17.6) | 3.4 (2.1-5.6) | 85.9 (80.7-89.9) | 5.6 (3.6-8.6) | 7.0 (4.1-11.5) | 87.9 (82.5-91.8) | 5.6 (3.6-8.7) | 6.1 (3.2-11.5) |
| Never married | 68.3 (62.2-73.9) | 9.8 (6.8-14.1) | 13.6 (9.8-18.5) | 85.5 (81.2-88.9) | 7.9 (5.7-10.8) | 2.0 (1.0-4.2) | 84.6 (79.1-88.8) | 9.1 (5.9-13.7) | 5.6 (3.2-9.8) | 78.8 (72.9-83.7) | 11.7 (8.0-16.8) | 8.2 (5.3-12.5) |
| Education |  |  |  |  |  |  |  |  |  |  |  |  |
| Less than high school | 69.5 (60.4-77.3) | 7.4 (3.8-13.7) | 7.0 (3.4-13.6) | 83.2 (74.8-89.1) | 2.6 (0.8-7.7) | 2.9 (1.0-8.0) | 86.1 (79.2-91.0) | 8.8 (4.8-15.3) | 5.1 (2.7-9.6) | 93.5 (88.0-96.5) | 3.0 (1.2-7.1) | 2.7 (1.0-7.1) |
| High school | 69.6 (62.9-75.5) | 8.1 (5.0-13.0) | 13.1 (9.1-18.6) | 79.1 (73.3-83.9) | 11.3 (7.9-15.9) | 4.1 (2.1-8.2) | 86.4 (81.2-90.4) | 6.4 (3.8-10.6) | 5.5 (3.0-9.6) | 85.7 (79.6-90.2) | 7.8 (4.5-13.2) | 5.4 (2.9-9.8) |
| Some college | 70.5 (66.2-74.5) | 11.6 (9.1-14.7) | 12.4 (9.7-15.8) | 80.6 (77.0-83.7) | 12.1 (9.8-14.8) | 4.2 (2.8-6.4) | 87.9 (84.4-90.6) | 6.1 (4.2-8.9) | 5.2 (3.5-7.7) | 85.5 (81.6-88.6) | 9.0 (6.5-12.3) | 5.2 (3.3-7.9) |
| Bachelor's degree | 78.9 (74.0-83.1) | 9.0 (6.3-12.8) | 7.8 (5.3-11.2) | 81.0 (76.9-84.5) | 12.5 (9.8-15.8) | 2.5 (1.6-3.8) | 85.9 (81.0-89.8) | 10.1 (6.6-15.0) | 2.8 (1.8-4.4) | 83.4 (79.0-87.0) | 11.0 (8.0-14.9) | 5.7 (3.7-8.4) |
| Advanced degree | 80.8 (76.1-84.7) | 9.5 (7.0-12.9) | 6.9 (4.6-10.3) | 78.6 (73.7-82.9) | 15.6 (11.8-20.3) | 3.5 (2.4-5.3) | 91.0 (87.5-93.6) | 5.3 (3.3-8.4) | 3.6 (2.2-5.9) | 85.7 (81.7-89.0) | 6.8 (4.5-10.1) | 6.7 (4.7-9.3) |
| Income |  |  |  |  |  |  |  |  |  |  |  |  |
| Less than $25,000 | 66.3 (59.0-73.0) | 8.0 (4.9-12.9) | 10.8 (7.3-15.8) | 78.7 (72.1-84.1) | 6.7 (4.5-10.1) | 3.8 (1.8-7.7) | 84.7 (78.8-89.2) | 7.8 (4.7-12.7) | 5.8 (3.3-10.0) | 87.3 (81.7-91.4) | 4.7 (2.7-8.1) | 6.7 (3.7-11.8) |
| $25,000-$59,999 | 69.8 (64.4-74.7) | 11.2 (8.2-15.2) | 9.6 (6.9-13.2) | 81.6 (76.9-85.5) | 8.7 (6.4-11.7) | 3.6 (1.8-6.9) | 86.9 (82.9-90.0) | 7.6 (5.1-11.1) | 4.7 (3.1-7.1) | 89.3 (85.3-92.3) | 5.8 (3.5-9.3) | 3.9 (2.4-6.4) |
| $60,000-$99,999 | 72.7 (67.3-77.5) | 10.0 (7.1-14.0) | 13.0 (9.5-17.6) | 78.7 (74.3-82.6) | 14.1 (11.0-17.9) | 4.6 (3.1-6.9) | 88.2 (83.7-91.6) | 7.0 (4.4-10.9) | 4.6 (2.7-7.8) | 86.5 (82.5-90.0) | 7.8 (5.4-11.2) | 5.4 (3.6-8.1) |
| $100,000 or more | 77.9 (73.8-81.6) | 8.7 (6.6-11.5) | 8.5 (6.1-11.7) | 81.5 (77.9-84.6) | 12.4 (10.0-15.2) | 3.0 (1.8-4.7) | 88.4 (84.9-91.1) | 6.8 (4.7-9.7) | 4.1 (2.6-6.3) | 84.2 (80.4-87.4) | 10.3 (7.7-13.7) | 5.1 (3.4-7.5) |

^a^Refusals and “don’t know” responses are not shown.

^b^ See “Methods” in supplement for exact text of questions.

**Supplemental Table 5. Total number of experiences of violence (EVs) by respondents’ marital status, education, and income^a^ (n=2870)**

| **Respondent Characteristic** | **Total number of reported EVs**  **Weighted % (95% CI)** | | | |
| --- | --- | --- | --- | --- |
|  | **0** | **1** | **2** | **≥3** |
| Marital status |  |  |  |  |
| Married | 39.1 (35.6-42.6) | 34.2 (31.0-37.8) | 16.3 (13.9-19.0) | 10.5 (8.5-12.9) |
| Widowed | 38.8 (29.8-48.6) | 34.0 (25.4-43.8) | 19.4 (12.4-29.0) | 7.8 (3.9-14.8) |
| Divorced | 37.1 (30.2-44.7) | 27.9 (22.5-34.0) | 20.2 (15.1-26.4) | 14.8 (10.0-21.3) |
| Never married | 24.3 (19.6-29.8) | 40.1 (34.0-46.5) | 22.8 (17.8-28.7) | 12.8 (9.4-17.3) |
| Education |  |  |  |  |
| Less than high school | 34.0 (26.2-42.9) | 37.4 (29.3-46.4) | 18.6 (12.5-26.7) | 9.9 (5.7-16.7) |
| High school | 29.3 (23.5-35.9) | 36.5 (30.2-43.3) | 21.8 (16.6-28.1) | 12.4 (8.5-17.7) |
| Some college | 33.9 (29.8-38.3) | 34.9 (30.8-39.3) | 18.0 (14.9-21.5) | 13.3 (10.4-16.8) |
| Bachelor's degree | 37.2 (31.9-42.7) | 35.4 (30.3-40.9) | 17.0 (13.2-21.7) | 10.4 (7.6-14.0) |
| Advanced degree | 46.8 (40.3-53.4) | 29.2 (23.9-35.0) | 15.8 (12.0-20.4) | 8.3 (6.1-11.2) |
| Income |  |  |  |  |
| Less than $25,000 | 29.3 (23.4-36.1) | 38.2 (31.4-45.5) | 19.6 (14.5-26.0) | 12.9 (8.9-18.2) |
| $25,000-$59,999 | 31.9 (27.0-37.1) | 35.5 (30.5-40.9) | 20.4 (16.5-25.1) | 12.2 (9.0-16.3) |
| $60,000-$99,999 | 33.3 (28.3-38.7) | 34.5 (29.4-40.0) | 19.3 (15.1-24.3) | 12.9 (9.5-17.3) |
| $100,000 or more | 41.1 (36.5-45.9) | 33.5 (29.2-38.0) | 16.1 (12.9-19.8) | 9.4 (7.0-12.5) |

^a^Refusals and “don’t know” responses are not shown.

**Supplemental Table 6. Combined social network experiences of violence by respondents’ age, race/ethnicity, gender, and firearm ownership (n= 2870)**

| **Respondent Characteristic** | **Number of known persons who have been shot or shot themselves on purpose**  **Weighted % (95% CI)** | | | | | **Number of known persons perceived to be at risk for violence**  **Weighted % (95% CI)** | | | | |
| --- | --- | --- | --- | --- | --- | --- | --- | --- | --- | --- |
|  |  | **Upper bound^a^** | | **Lower bound^a^** | |  | **Upper bound^a^** | | **Lower bound^a^** | |
|  | **0** | **1** | **≥2** | **1** | **≥2** | **0** | **1** | **≥2** | **1** | **≥2** |
| Age |  |  |  |  |  |  |  |  |  |  |
| 18-29 | 68.6 (60.5-75.7) | 11.4 (7.4-17.1) | 17.1 (11.6-24.5) | 12.8 (8.6-18.6) | 15.7 (10.3-23.1) | 66.9 (58.5-74.4) | 15.8 (10.4-23.4) | 16.5 (11.2-23.6) | 16.7 (11.1-24.5) | 15.6 (10.5-22.5) |
| 30-44 | 67.8 (62.1-72.9) | 10.7 (7.9-14.5) | 14.6 (11.1-19.0) | 12.0 (9.0-15.8) | 13.3 (9.9-17.7) | 77.1 (72.2-81.4) | 11.6 (8.6-15.6) | 10.4 (7.6-14.0) | 13.2 (9.9-17.1) | 8.8 (6.3-12.2) |
| 45-59 | 64.7 (59.8-69.4) | 14.4 (11.2-18.2) | 18.3 (14.7-22.5) | 17.5 (14.0-21.7) | 15.1 (11.9-19.0) | 79.8 (75.5-83.6) | 11.2 (8.3-14.8) | 8.5 (6.1-11.8) | 13.0 (9.8-17.4) | 6.7 (4.8-9.3) |
| 60+ | 68.9 (65.5-72.1) | 19.0 (16.4-21.9) | 11.4 (9.4-13.7) | 21.3 (18.5-24.3) | 9.1 (7.3-11.2) | 85.4 (83.0-87.6) | 7.8 (6.3-9.5) | 6.8 (5.3-8.7) | 8.7 (7.1-10.7) | 5.8 (4.4-7.6) |
| Race/ethnicity |  |  |  |  |  |  |  |  |  |  |
| White | 66.6 (63.0-69.9) | 18.5 (16.0-21.2) | 13.4 (11.0-16.1) | 20.7 (18.1-23.6) | 11.1 (8.9-13.8) | 76.6 (73.3-79.7) | 11.5 (9.4-14.1) | 11.3 (9.1-14.0) | 13.1 (10.8-15.8) | 9.7 (7.6-12.2) |
| Black | 50.2 (38.3-62.1) | 10.6 (5.5-19.5) | 34.2 (23.8-46.3) | 12.4 (6.8-21.3) | 32.4 (22.2-44.6) | 85.7 (74.9-92.3) | 10.5 (4.7-21.8) | 3.9 (1.8-8.3) | 10.5 (4.7-21.8) | 3.9 (1.8-8.3) |
| Asian American | 82.3 (74.9-87.8) | 10.2 (6.3-16.2) | 6.6 (3.3-12.8) | 11.7 (7.5-17.,9) | 5.2 (2.3-11.4) | 81.9 (74.0-87.8) | 12.7 (7.9-19.8) | 4.7 (2.0-10.8) | 15.6 (10.0-23.4) | 1.8 (0.7-5.0) |
| Multiracial or Other | 66.1 (48.0-80.4) | 9.7 (4.0-21.8) | 23.1 (10.9-42.6) | 11.9 (5.5-23.7) | 21.0 (9.2-41.2) | 65.7 (47.3-80.4) | 19.3 (8.5-38.1) | 14.9 (6.0-32.6) | 19.7 (8.9-38.3) | 14.5 (5.7-32.4) |
| Latinx | 65.2 (60.3-69.8) | 11.3 (8.6-14.6) | 16.8 (13.6-20.7) | 13.5 (10.6-17.1) | 14.7 (11.6-18.3) | 79.2 (74.9-82.9) | 9.4 (6.8-12.7) | 10.9 (8.3-14.3) | 10.2 (7.5-13.6) | 10.1 (7.6-13.4) |
| Gender |  |  |  |  |  |  |  |  |  |  |
| Male | 68.1 (64.2-71.7) | 12.3 (10.1-14.9) | 15.4 (12.8-18.5) | 13.6 (11.3-16.2) | 14.1 (11.6-17.1) | 79.2 (75.6-82.3) | 11.4 (8.9-14.4) | 8.6 (6.6-11.0) | 12.7 (10.1-15.8) | 7.3 (5.6-9.5) |
| Female | 66.8 (63.1-70.2) | 15.7 (13.3-18.4) | 14.8 (12.3-17.8) | 18.5 (15.9-21.4) | 12.0 (9.7-14.9) | 77.8 (74.5-80.8) | 10.9 (8.8-13.5) | 11.1 (8.9-13.8) | 12.3 (10.0-15.1) | 9.7 (7.6-12.2) |
| Household firearm ownership |  |  |  |  |  |  |  |  |  |  |
| Non-owner | 70.6 (67.5-73.5) | 12.9 (11.0-14.9) | 13.0 (10.9-15.3) | 14.8 (12.9-17.1) | 11.0 (9.1-13.2) | 79.8 (77.0-82.3) | 11.0 (9.1-13.3) | 8.9 (7.3-10.9) | 12.4 (10.4-14.9) | 7.5 (6.0-9.2) |
| Firearm owner | 61.5 (54.9-67.7) | 16.3 (12.3-21.3) | 20.7 (16.0-26.4) | 19,4 (15.1-24.6) | 17.6 (13.1-23.1) | 81.8 (76.3-86.2) | 9.4 (6.6-13.1) | 8.8 (5.5-13.9) | 11.3 (8.0-15.8) | 6.9 (4.2-11.2) |
| Non-owner living with owner | 56.7 (46.8-66.0) | 18.6 (12.4-27.0) | 24.3 (16.6-34.2) | 21.0 (14.4-29.4) | 22.0 (14.5-32.0) | 62.6 (52.4-71.8) | 16.9 (10.3-26.5) | 20.6 (13.5-30.0) | 17.6 (10.9-27.1) | 19.9 (12.9-29.4) |

^a^The upper bound estimate makes no allowance for duplication and is based on the sum of known persons reported across multiple social network experiences. The lower bound estimate assumes complete duplication and is based on the largest number of known persons reported for any 1 of those experiences.

**Supplemental Table 7. Combined social network experiences of violence by respondents’ marital status, education, and income (n= 2870)**

| **Respondent Characteristic** | **Number of known persons who have been shot or shot themselves on purpose Weighted % (95% CI)** | | | | | **Number of known persons perceived to be at risk for violence**  **Weighted % (95% CI)** | | | | |
| --- | --- | --- | --- | --- | --- | --- | --- | --- | --- | --- |
|  |  | **Upper bound^a^** | | **Lower bound^a^** | |  | **Upper bound^a^** | | **Lower bound^a^** | |
|  | **0** | **1** | **≥2** | **1** | **≥2** | **0** | **1** | **≥2** | **1** | **≥2** |
| Marital status |  |  |  |  |  |  |  |  |  |  |
| Married | 68.9 (65.6-72.1) | 13.1 (11.2-15.3) | 14.4 (12.1-17.1) | 15.2 (13.1-17.5 | 12.3 (10.2-14.9) | 81.5 (78.7-84.0) | 10.1 (8.2-12.4) | 8.1 (6.5-10.0) | 11.6 (9.5-14.0) | 6.6 (5.3-8.3) |
| Widowed | 63.8 (53.8-72.8) | 21.0 (14.0-30.5) | 14.4 (8.6-23.1) | 25.3 (17.5-35.0) | 10.2 (5.4-18.3) | 86.4 (78.9-91.5) | 8.6-(4.8-15.0) | 4.9 (2.0-11.1) | 8.7 (4.9-15.1) | 4.8 (2.0-11.1) |
| Divorced | 62.5 (55.3-69.3) | 17.9 (12.9-24.2) | 16.1 (11.5-22.2) | 20.6 (15.5-27.0) | 13.4 (9.0-19.5) | 76.1 (69.7-81.6) | 10.1 (7.2-14.0) | 12.6 (8.3-18.6) | 10.6 (7.6-14.5) | 12.1 (7.9-18.2) |
| Never married | 66.4 (60.3-72.0) | 13.5 (9.9-18.1) | 16.5 (12.5-21.6) | 14.8 (11.2-19.4) | 15.2 (11.3-20.2) | 70.3 (64.0-75.9) | 14.7 (10.5-20.3) | 14.3 (10.3-19.5) | 16.5 (12.1-22.3) | 12.5 (8.8-17.4) |
| Education |  |  |  |  |  |  |  |  |  |  |
| Less than high school | 72.8 (63.7-80.2) | 6.9 (3.7-12.5) | 10.8 (6.1-18.3) | 8.1 (4.4-14.4) | 9.5 (5.3-16.6) | 83.6 (76.4-88.9) | 9.1 (5.2-15.4) | 7.3 (4.1-12.6) | 9.9 (5.9-16.4) | 6.5 (3.6-11.5) |
| High school | 64.4 (57.7-70.6) | 13.7 (9.8-18.7) | 17.4 (12.7-23.3) | 14.5 (10.6-19.6) | 16.5 (12.0-22.4) | 76.3 (69.6-81.9) | 11.8 7.7-17.7) | 10.9 (7.2-16.1) | 12.8 (8.5-18.7) | 9.9 (6.4-15.1) |
| Some college | 63.9 (59.6-68.1) | 16.5 (13.6-19.7) | 17.9 (14.7-21.7) | 18.8 (15.8-22.3) | 15.6 (12.5-19.2) | 79.1 (75.1-82.7) | 9.8 (7.4-12.8) | 10.8 (8.0-14.2) | 11.7 (8.9-15.0) | 8.9 (6.5-12.0) |
| Bachelor's degree | 70.4 (65.3 (75.0) | 15.2 (11.8-19.5) | 12.2 (9.3-15.8) | 17.3 (13.7-21.6) | 10.0 (7.4-13.5) | 73.4 (68.1-78.2) | 15.3 (11.5-20.1) | 10.3 (7.4-14.0) | 17.4 (13.3-22.6) | 8.1 (5.9-11.1) |
| Advanced degree | 70.3 (64.8-75.3) | 15.5 (11.9-20.0) | 13.7 (10.4-17.8) | 19.5 (15.4-24.4) | 9.7 (7.0-13.1) | 80.7 (76.1-84.6) | 10.3 (7.3-14.4) | 8.9 (6.6-11.9) | 10.7 (7.7-14.8) | 8.5 (6.2-11.4) |
| Income |  |  |  |  |  |  |  |  |  |  |
| Less than $25,000 | 64.7 (57.4-71.3) | 12.0 (8.2-17.2) | 14.3 (10.1-19.8) | 12.6 (8.7-17.8) | 13.7 (9.6-19.2) | 78.1 (71.7-83.5) | 9.1 (6.0-13.7) | 11.5 (7.5-17.1) | 10.7 (7.2-15.7) | 9.8 (6.2-15.3) |
| $25,000-$59,999 | 67.5 (62.2-72.3) | 14.4 (11.3-18.2) | 14.2 (10.8-18.5) | 16.0 (12.8-19.9) | 12.6 (9.3-16.9) | 80.8 (76.2-84.7) | 10.5 (7.5-14.4) | 8.7 (6.2-12.0) | 11.5 (8.3-15.6) | 7.6 (5.4-10.8) |
| $60,000-$99,999 | 65.3 (59.9-70.3) | 14.4 (11.2-18.3) | 19.3 (15.2-24.2) | 17.7 (14.1-22.1) | 16.0 (12.2-20.6) | 77.6 (72.5-82.0) | 12.1 (8.7-16.6) | 10.2 (7.4-14.0) | 12.8 (9.3-17.3) | 9.6 (6.8-13.3) |
| $100,000 or more | 69.6 (65.3-73.7) | 14.4 (11.7-17.6) | 13.6 (10.7-17.0) | 16.7 (13.8-20.0) | 11.3 (8.6-14.7) | 77.6 (73.5-81.3) | 11.8 (9.0-15.2) | 9.9 (7.4-13.0) | 13.7 (10.7-17.4) | 7.9 (5.8-10.7) |

^a^The upper bound estimate makes no allowance for duplication and is based on the sum of known persons reported across multiple social network experiences. The lower bound estimate assumes complete duplication and is based on the largest number of known persons reported for any 1 of those experiences.

**Supplemental Table 8. Total social network experiences of violence by respondents’ age, race/ethnicity, gender, and firearm ownership (n= 2870)**

| **Respondent Characteristic** | **Number of known persons who have been shot or shot themselves on purpose or are perceived to be at risk for violence**  **Weighted % (95% CI)** | | | | |
| --- | --- | --- | --- | --- | --- |
|  |  | **Upper bound^a^** | | **Lower bound^a^** | |
|  | **0** | **1** | **≥2** | **1** | **≥2** |
| Age |  |  |  |  |  |
| 18-29 | 50.8 (42.4-59.2) | 18.1 (12.3-25.7) | 30.9 (23.7-39.1) | 20.4 (14.4-28.1) | 28.5 (21.5-36.7) |
| 30-44 | 61.5 (55.9-66.9) | 13.5 (10.3-17.6) | 24.2 (19.8-29.3) | 17.8 (14.1-22.3) | 19.9 (15.8-24.7) |
| 45-59 | 57.9 (52.8-62.8) | 15.6 (12.3-19.5) | 26.5 (22.3-31.2) | 22.1 (18.2-26.7) | 20.0 (16.3-24.2) |
| 60+ | 61.6 (58.0-65.0) | 19.8 (17.1-22.8) | 18.6 (16.2-21.4) | 24.6 (21.6-27.7) | 13.9 (11.7-16.3) |
| Race/ethnicity |  |  |  |  |  |
| White | 55.6 (51.9-59.3) | 18.7 (16.1-21.6) | 25.4 (22.3-28.8) | 24.6 (21.7-27.8) | 19.5 (16.6-22.8) |
| Black | 51.6 (39.7-63.4) | 12.7 (7.0-21.8) | 35.7 (25.2-47.8) | 14.5 (8.4-23.7) | 33.9 (23.6-46.1) |
| Asian American | 73.4 (65.2-80.3) | 15.9 (10.6-23.2) | 10.7 (6.4-17.3) | 19.9 (13.9-27.5) | 6.7 (3.4-12.9) |
| Multiracial or Other | 46.8 (30.6-63.7) | 25.9 (13.4-44.2) | 27.3 (14.3-45.7) | 28.5 (15.1-46.3) | 24.7 (12.2-43.7) |
| Latinx | 59.0 (54.1-63.7) | 13.9 (10.9-17.5) | 26.7 (22.6-31.2) | 18.2 (14.7-22.2) | 22.4 (18.6-26.7) |
| Gender |  |  |  |  |  |
| Male | 60.8 (56.8-64.6) | 14.6 (12.0-17.5) | 24.1 (20.9-27.6) | 18.9 (16.1-22.2) | 19.7 (16.8-23.1) |
| Female | 57.1 (53.3-60.8) | 18.3 (15.6-21.3) | 24.7 (21.5-28.1) | 23.3 (20.4-26.6) | 19.6 (16.7-22.9) |
| Household firearm ownership |  |  |  |  |  |
| Non-owner | 62.0 (58.8-65.1) | 16.3 (14.0-18.8) | 21.7 (19.2-24.5) | 20.9 (18.4-23.7) | 17.1 (14.8-19.7) |
| Firearm owner | 54.8 (48.2-61.3) | 16.8 (13.0-21.4) | 28.4 (22.9-34.6) | 21.8 (17.5-26.8) | 23.4 (18.2-29.5) |
| Non-owner living with owner | 42.1 (32.9-51.9) | 16.7 (11.2-24.1) | 41.3 (31.8-51.4) | 24.0 (16.9-32.8) | 33.9 (25.1-44.1) |

^a^The upper bound estimate makes no allowance for duplication and is based on the sum of known persons reported across the 4 social network experiences. The lower bound estimate assumes complete duplication and is based on the largest number of known persons reported for any 1 of those experiences.

**Supplemental Table 9. Total social network experiences of violence by respondents’ marital status, education, and income (n= 2870)**

| **Respondent Characteristic** | **Number of known persons who have been shot or shot themselves on purpose or are perceived to be at risk for violence**  **Weighted % (95% CI)** | | | | |
| --- | --- | --- | --- | --- | --- |
|  |  | **Upper bound^a^** | | **Lower bound^a^** | |
|  | **0** | **1** | **≥2** | **1** | **≥2** |
| Marital status |  |  |  |  |  |
| Married | 62.6 (59.2-65.9) | 15.0 (12.9-17.5) | 22.1 (19.4-25.2) | 19.7 (17.2-22.4) | 17.5 (15.0-20.3) |
| Widowed | 58.0 (48.1-67.4) | 23.3 (15.7-33.1) | 18.7 (12.3-27.3) | 29.7 (21.4-39.6) | 12.3 (7.1-20.3) |
| Divorced | 54.2 (46.9-61.2) | 17.7 (13.3-23.1) | 28.2 (22.0-35.3) | 21.8 (17.0-27.6) | 24.0 (18.0-31.2) |
| Never married | 51.3 (45.0-57.6) | 18.5 (13.9-24.2) | 29.5 (24.1-35.5) | 23.4 (18.4-29.3) | 24.6 (19.6-30.4) |
| Education |  |  |  |  |  |
| Less than high school | 68.5 (59.8-76.0) | 12.9 (8.3-19.4) | 18.7 (12.6-26.8) | 16.1 (10.7-23.6) | 15.4 (10.1-22.9) |
| High school | 54.8 (47.9-61.5) | 16.9 (12.2-22.9) | 27.6 (21.8-34.2) | 20.1 (15.1-26.2) | 24.4 (18.8-31.0) |
| Some college | 56.2 (51.8-60.6) | 15.7 (12.9-19.0) | 27.7 (23.8-32.0) | 21.9 (18.6-25.6) | 21.6 (18.0-25.6) |
| Bachelor's degree | 57.6 (52.1-62.9) | 20.6 (16.3-25.6) | 21.8 (17.9-26.3) | 25.5 (21.0-30.7) | 16.9 (13.4-21.1) |
| Advanced degree | 61.8 (55.8-67.5) | 16.7 (12.9-21.3) | 21.5 (17.4-26.4) | 21.6 (17.2-26.8) | 16.6 (13.1-20.7) |
| Income |  |  |  |  |  |
| Less than $25,000 | 60.2 (53.1-66.9) | 13.4 (9.4-18.7) | 25.3 (19.7-32.0) | 16.6 (12.2-22.2) | 22.1 (16.7-28.7) |
| $25,000-$59,999 | 61.6 (56.3-66.7) | 14.7 (11.6-18.6) | 23.6 (19.3-28.6) | 19.8 (16.0-24.2) | 18.6 (14.7-23.2) |
| $60,000-$99,999 | 52.9 (47.3-58.5) | 18.6 (14.7-23.2) | 28.5 (23.6-33.9) | 23.9 (19.5-29.0) | 23.1 (18.7-28.3) |
| $100,000 or more | 60.0 (55.4-64.5) | 17.6 (14.4-21.4) | 22.1 (18.6-26.1) | 22.4 (18.8-26.3) | 17.3 (14.1-21.1) |

^a^The upper bound estimate makes no allowance for duplication and is based on the sum of known persons reported across the 4 social network experiences. The lower bound estimate assumes complete duplication and is based on the largest number of known persons reported for any 1 of those experiences.
